# In host evolution of beta lactam resistance during active treatment for *Pseudomonas aeruginosa* bacteremia

**DOI:** 10.3389/fcimb.2023.1241608

**Published:** 2023-08-30

**Authors:** Natasha Spottiswoode, Samantha Hao, Estella Sanchez-Guerrero, Angela M. Detweiler, Honey Mekonen, Norma Neff, Henriette Macmillan, Brian S. Schwartz, Joanne Engel, Joseph L. DeRisi, Steven A. Miller, Charles R. Langelier

**Affiliations:** ^1^ Division of Infectious Diseases, Department of Medicine, University of California, San Francisco, San Francisco, CA, United States; ^2^ Johns Hopkins School of Medicine, Baltimore, Maryland, MD, United States; ^3^ Chan Zuckerberg Biohub, San Francisco, CA, United States; ^4^ Department of Biochemistry and Biophysics, University of California, San Francisco, San Francisco, CA, United States; ^5^ Department of Laboratory Medicine, University of California, San Francisco, San Francisco, CA, United States; ^6^ Delve Bio Inc., San Francisco, CA, United States

**Keywords:** antimicrobial resistance, whole-genome sequencing, gram-negative bacteria, gram negative, gram negative (G -) bacteria, hospital epidemiology

## Abstract

Multidrug-resistant (MDR) *Pseudomonas aeruginosa* has been declared a serious threat by the United States Centers for Disease Control and Prevention. Here, we used whole genome sequencing (WGS) to investigate recurrent *P. aeruginosa* bloodstream infections in a severely immunocompromised patient. The infections demonstrated unusual, progressive increases in resistance to beta lactam antibiotics in the setting of active treatment with appropriate, guideline-directed agents. WGS followed by comparative genomic analysis of isolates collected over 44 days demonstrated in host evolution of a single *P. aeruginosa* isolate characterized by stepwise acquisition of two *de-novo* genetic resistance mechanisms over the course of treatment. We found a novel deletion affecting the *ampC* repressor *ampD* and neighboring gene *ampE*, which associated with initial cefepime treatment failure. This was followed by acquisition of a porin nonsense mutation, *OprD*, associated with resistance to carbapenems. This study highlights the potential for in-host evolution of *P. aeruginosa* during bloodstream infections in severely immunocompromised patients despite appropriate antimicrobial therapy. In addition, it demonstrates the utility of WGS for understanding unusual resistance patterns in the clinical context.

## Manuscript contribution to the field

Multidrug-resistant (MDR) *Pseudomonas aeruginosa* is an increasing threat world-wide. To anticipate and treat MDR bacterial infections, an in-depth understanding of resistance acquisition in clinical settings is paramount. In this case, we used serial genomic analysis to study in-host evolution of a recurrent *P. aeruginosa* bloodstream infection in a severely immunocompromised patient. We found that infections were caused by the same strain of MDR *P. aeruginosa* which acquired *de-novo* resistance to increasingly broad spectrum, guideline-directed, anti-pseudomonal antibiotics through serial acquisition of a previously undescribed *ampD* deletion, and a porin mutation, respectively. This study demonstrates both the potential for in-host evolution of bacterial pathogens in immunocompromised hosts undergoing antimicrobial therapy, and highlights the utility of clinical WGS in understanding MDR organism epidemiology and in-host evolution.

## Introduction

Multidrug resistant (MDR) Pseudomonas aeruginosa, as defined by resistance to three or more of the major anti-pseudomonal antibiotic classes, is a global health threat of increasing importance (Magiorakos et al., 2012; CDC, 2019). *P. aeruginosa* can cause a multitude of infectious syndromes, including pneumonia, bloodstream infections, and wound infections. *P. aeruginosa* is also an infamous cause of nosocomial infections, because it can transmit effectively within the hospital environment, by contaminating surfaces such as sinks, ultrasound gel, endoscopes, or beds; it is secondary only to *Acinetobacter baumanii* (also designed a top threat by the CDC) in its ability to contaminate healthcare workers’ gowns and gloves (Morgan et al., 2012). Complementing this ability to transmit in-hospital, *P. aeruginosa* has a remarkable propensity to acquire resistance through multiple mechanisms, including alteration of porins, efflux pumps, acquisition of beta-lactamases such as *ampC* or extended-spectrum beta-lactamases (ESBL), carbapenemases, and aminoglycoside nucleotidyl-transferases. Recognizing this growing threat, the Infectious Disease Society recently released an updated dedicated guideline document recommending standardized treatment of treatment of MDR *P. aeruginosa* and other highly resistant gram-negative bacterial organisms (Tamma et al, 2023).

Key to combating MDR *P. aeruginosa* is a detailed understanding of its epidemiology and mechanisms of resistance. Patients may acquire MDR *P. aeruginosa* infection endogenously by in-host evolution of *P. aeruginosa* under antibiotic pressure, or exogenous infection with MDR *P. aeruginosa* from another patient, healthcare-workers, or the healthcare environment (Johnson et al., 2009). A study of the acquisition of imipenem-resistant *P. aeruginosa* in intensive care units between 2001-2006 noted that approximately 50% of imipenem-resistant *P. aeruginosa* cases were linked to in-patient evolution and 50% were directly acquired from another patient or the hospital environment (Morgan et al., 2012).

The use of whole-genome sequencing (WGS) as part of hospital epidemiology and outbreak investigations holds considerable promise. Our hospital routinely performs WGS to evaluate suspected healthcare transmission of microbes (Crawford et al., 2020), track newly emerging pathogens (Woodworth et al., 2019), and analyze cases of unusual antimicrobial resistance patterns (Hao et al., 2020a). Here, we performed WGS of serially collected *P. aeruginosa* isolates to study progressive acquisition of resistance despite guideline-directed antimicrobial treatment in an immunocompromised host. We found that in-host evolution rather than exogenous acquisition of an antimicrobial resistance gene was responsible, and involved a novel deletion in *ampD* and *ampE*, and a mutation in the porin gene *OprD.*

## Methods

### Sample collection protocol

Patient blood cultures were performed via inoculation into BD Bactec Plus Aerobic and Lytic Anaerobic media (Becton Dickinson). Species identification was performed using matrix-assisted laser desorption/ionization time of-flight mass spectrometry (Bruker). Minimum inhibitory concentrations (MICs) were determined using the Trek Sensititre automated broth microdilution system and the Clinical and Laboratory Standards Institute (CLSI) breakpoints.

### Ethics Statement

Investigations were carried out according to a no-subject contact study protocol approved by the University of California San Francisco Institutional Review Board, which permitted analysis of deidentified leftover clinical microbiology samples from collaborating institutions and subsequent review of study participants’ electronic medical records. No decisions regarding antibiotics or other patient-specific treatment interventions were made using sequencing data.

### Whole genome sequencing

Whole genome sequencing was performed according to a previously described protocol (Crawford et al., 2020). Briefly, DNA from each sample was sheared with fragmentase (New England Biolabs) and used to construct sequencing libraries with the NEBNext Ultra II Library Prep Kit (New England Biolabs). Adaptor ligated samples underwent amplification with dual unique indexing primers. Libraries were quantified and pooled and underwent paired end 150 base pair sequencing on an Illumina MiSeq.

### Bioinformatics and phylogenetic analyses

Phylogenetic analyses were performed according to established methods (Hao et al., 2020b). Briefly, short reads were adapter trimmed, quality filtered with fastp v0.20.0, and analyzed using the core single-nucleotide polymorphism (SNP) detection pipeline SPID v0.4.0 (https://github.com/czbiohub/Spid.jl). Genome assembly was performed using Unicycler v0.4.8 (Wick et al., 2017), and BLAST of the largest contig identified *P. aeruginosa* strain F9676 (Genbank number CP012066.1) as the most closely related strain to serve a reference genome (Chen et al., 2018). INDEL analysis was performed with Snippy (Seemann, 2018). Finally, the maximum likelihood phylogeny based on the SNP alignments was built in RAxML v8.2.12 (Stamatakis, 2014).

## Results

**Figure 1:**
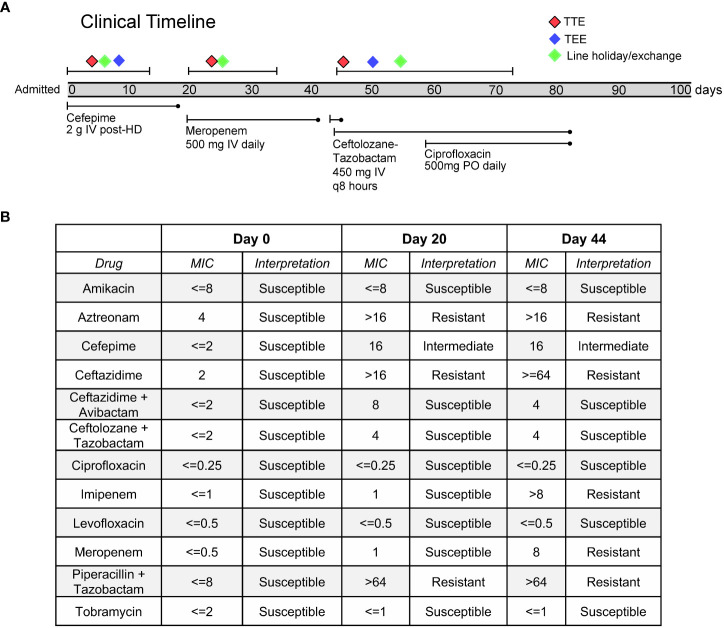
**(A)** Timeline of clinical case and evolution of antibiotic resistance in the *Pseudomonas aeruginosa.* **(B)** Phenotypic antimicrobial susceptibility testing of clinical blood culture isolates.

A 55-year-old woman with a history of B-cell lymphoma, allogeneic stem cell transplant complicated by graft versus host disease, and renal failure on intermittent hemodialysis, was admitted for sepsis secondary to pan-susceptible *P. aeruginosa* bacteremia (**Figure 1**). Direct time to positivity (DTTP) blood cultures drawn through her tunneled dialysis catheter (TDC) and peripherally inserted central catheter (PICC) did not implicate the lines as the source of infection. Both lines were removed on day five, after which her cultures cleared. A transthoracic echocardiogram demonstrated no evidence of endocarditis, and a transesophageal echocardiogram (TEE) demonstrated mitral valve thickening without vegetation. New PICC and TDC lines were placed after 48 hours, and she was discharged on two weeks of cefepime for treatment of bacteremia (**Figure 1**).

One day after completing her cefepime regimen, she developed fevers and re-presented to the hospital, where blood cultures drawn from her PICC, TDC, and peripheral blood again grew *P. aeruginosa*, but now with new intermediate resistance to cefepime, as well as resistance to aztreonam, piperacillin-tazobactam and ceftazidime. Even though DTTP blood cultures did not clearly implicate the TDC and PICC as sources of infection, both lines were again removed and later replaced, and she was prescribed a two-week course of meropenem starting from the date of culture clearance. Following clinical improvement, she was discharged home.

Two days after completion of her course of meropenem, the patient developed fevers, chills, and right upper quadrant pain. She re-presented to the hospital for a third time and was found to be growing *P. aeruginosa* from peripheral blood cultures, and the isolate was now additionally resistant to meropenem. She was started on ceftolozane-tazobactam. A repeat TEE was performed and now revealed a small mobile echodensity on the anterior leaflet of the mitral valve, thought to represent a possible vegetation. An abdominal CT scan demonstrated hepatic micro abscesses that were not amenable to drainage.

**Figure 2:**
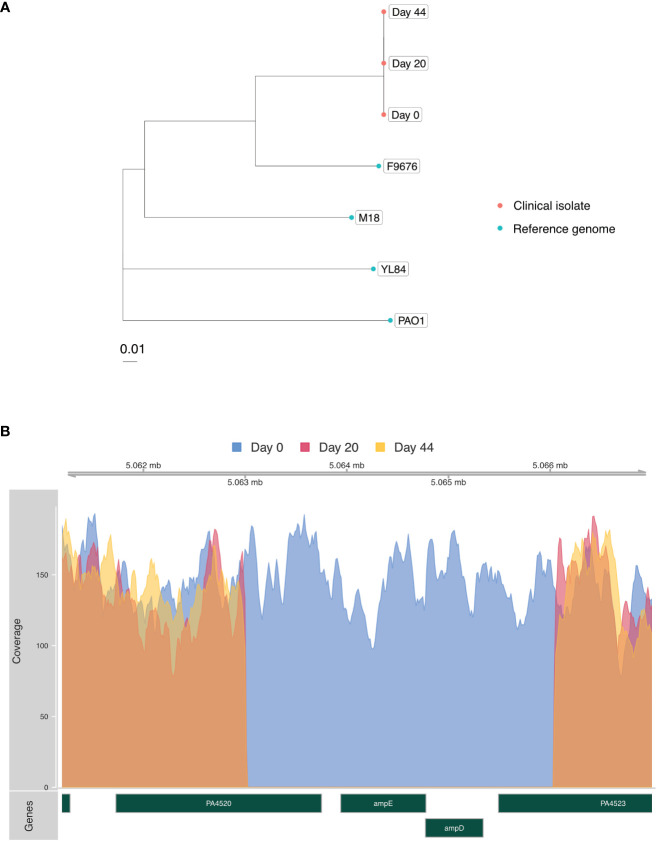
**(A)** Phylogenetic tree built using RAxML (Stamatakis, 2014) and the SNP Pipeline for Infectious Diseases (SPID) (Hao et al., 2020b) demonstrating genetic relatedness of the three *P. aeruginosa* isolates (red) from the patient in relation to reference genomes from NCBI Genbank (blue). Scale bar indicates substitutions per site. **(B)** Coverage plot of the *P. aeruginosa* genome assembled from clinical isolates highlighting a 3,794 base pair deletion involving the *ampD* and *ampE* genes present in the day-20 and day-44 isolates. Acquisition of this deletion, functionally predicted to de-repress the *ampC* beta lactamase gene, coincided with development of cefepime resistance and clinical treatment failure. rpm = *P. aeruginosa* reads per million reads sequenced.

In an effort to determine whether her multiple episodes of *P. aeruginosa* bacteremia with progressively increased drug resistance were due to independent infection events or due to evolution of resistance within a single strain, Illumina whole-genome sequencing (WGS) was performed on DNA extracted from blood culture isolates obtained on days 0, 20 and 44, according to an established protocol (Crawford et al., 2020) (**Figure 1**). Phylogenetic analysis demonstrated that all isolates represented the same strain, differing by a maximum of 3 single nucleotide polymorphisms (SNPs) across a core shared genome of 6,368,008 nucleotides (**Figure 2A**). This confirmed that resistance had progressively evolved, and ruled-out *de novo* acquisition of independent infections. Prediction of multilocus sequence type (MLST) from WGS data demonstrated that the isolate was most closely related to sequence type 167, and thus differed from several well-known high-risk international strains (Jolley et al., 2018).

Interrogation of the WGS data was then carried out to identify a molecular explanation for the observed resistance. Intriguingly, a 3,794 base pair deletion involving *ampD* and *ampE* was identified in isolates from days 20 and 44 (**Figure 2B**) that coincided with acquisition of new beta lactam resistance and cefepime clinical treatment failure. AmpD represses the expression of the *ampC* beta lactamase gene, and *in vitro* deletions in *ampD* have been associated with augmented expression of *ampC* and increased beta lactam resistance (Zamorano et al., 2010). Only one prior case of *P. aeruginosa* resistance attributed to an *ampD* mutation has been reported, and this involved an *ampD* missense mutation implicated in the rapid acquisition of resistance to multiple beta-lactam antibiotics (Barbosa et al., 2020).

In addition to the novel deletion, the blood culture isolate from day 44 was also found to contain a C748T nonsense mutation in *OprD*, paralleling the acquisition of carbapenem resistance during the patient’s secondary treatment regimen with meropenem. *OprD* encodes a 443-amino acid porin that forms a monomeric 18-stranded β barrel with 9 loops; it interacts with carbapenems and permits their entrance to the cell (Li et al., 2012; Jumper et al., 2021; Varadi et al., 2022). The mutation we identified, C748T, would be predicted to generate a truncated 249-amino acid protein that would most likely be non-functional. Prior work has associated other *OprD* mutations with carbapenem resistance (Marvig et al., 2013; Wheatley et al., 2021). Increased resistance to ceftazidime also characterized the day 44 isolate, which could potentially be due to OprD loss of function, which has been associated with increased resistance to ceftazidime in a large study of clinical isolates (Castanheira et al., 2014).

The patient was ultimately treated for endocarditis with four weeks of ceftolozane-tazobactam plus ciprofloxacin, according to clinical guidelines (Baddour et al., 2015). Following completion of therapy, the patient has not experienced any further recurrent episodes of bacteremia due to MDR *P. aeruginosa*.

## Discussion

MDR *P. aeruginosa* infections can arise in the hospital from direct human to human transmission, environmental acquisition, or from endogenous evolution in the setting of antibiotic selective pressure. While the latter appears to be the least common, it may account for as many as 19% of hospital-onset carbapenem-resistant *P. aeruginosa* infections (Johnson et al., 2009). The progressive acquisition of increasing resistance in the setting of appropriate anti-pseudomonal antibiotics, however, has not been widely reported. Reflecting this, guidelines recommend a non-carbapenem anti-pseudomonal agent as a first-line approach for susceptible isolates (Tamma et al., 2023).

In this case, initial imaging did not demonstrate discrete foci of infection amenable to source control, or an intravascular infection. It is possible that clinically occult endocarditis or an abdominal infection could have been present at the time of the patient’s initial hospitalization, but was too subtle to be confidently detected by echocardiography or CT scan. It is perhaps more likely that the patient’s underlying immunocompromised state impaired effective clearance of the pathogen, facilitating *de-novo* evolution of resistance. This phenomenon has been recently well described in the setting of SARS-CoV-2 respiratory infections in severely immunocompromised individuals (Scherer et al., 2022), but is not well described in the setting of bacteremia.

WGS is increasingly becoming a tool of choice for understanding the epidemiology and drug resistance mechanisms of MDR *P. aeruginosa* (Sundermann et al., 2021). While WGS is an established tool for outbreak investigation and pathogen surveillance (Stanton et al., 2022), its use to inform care for individual patient cases has been more limited. Several case studies have used WGS to identify the mechanisms underlying *P. aeruginosa* drug resistance in single patients (Alamri et al., 2020; Goldenberger et al., 2020; Hao et al., 2021). Few studies, however, have longitudinally assessed in host evolution using WGS, and those to date have focused exclusively on pulmonary infections (Datar et al., 2021; Wheatley et al., 2021; Rojas et al., 2022). An important recent study described the progressive acquisition, and loss, of carbapenem resistance during a prolonged *P. aeruginosa* pulmonary infection associated with the acquisition of mutations in *oprD* and other genes (Wheatley et al., 2021).

This study extends prior *in vivo* longitudinal genomic analyses of this pathogen to the setting of bacterial bloodstream infections. Furthermore, it highlights the potential clinical utility of WGS for understanding progressive and unexplained drug resistance during treatment, and for disambiguating hospital-acquired infections from cases of in-host evolution. Clinical deployment of WGS is not yet widely available, but may soon offer a sufficiently rapid turn-around time to directly impact clinical care.

## Data availability statement

The datasets presented in this study can be found in online repositories. The names of the repository/repositories and accession number(s) can be found below: https://www.ncbi.nlm.nih.gov/, PRJNA888413.

## Ethics statement

The studies involving humans were approved by University of California San Francisco Institutional Review Board. The studies were conducted in accordance with the local legislation and institutional requirements. The human samples used in this study were acquired from a by- product of routine care or industry. Written informed consent for participation was not required from the participants or the participants’ legal guardians/next of kin in accordance with the national legislation and institutional requirements.

## Author contributions

NS, SH, and EG: Equal contributions. NS lead writing, clinical data review and integration. SH lead genomic and computational analyses. EG lead microbiology and whole genome sequencing. AD, HMe, NN, and HMa provided computational analysis and pipeline support. BS and JE advised clinical data review and microbiological interpretation. JLD, SM, and CL oversaw all aspects of study and writing. All authors contributed to the article and approved the submitted version.
